# Supplementary material for: A public health community health worker-delivered intervention to reduce human trafficking among Denotified Tribes in India: A protocol paper
Source: PLoS One. 2025 Mar 13;20(3):e0317733. doi: 10.1371/journal.pone.0317733 (PMC11906080; doi:10.1371/journal.pone.0317733)
Supplement: Attachment — Submitted filename: PONE-D-24-33545_rev2comments.docx [file pone.0317733.s001.docx]

**PLOS ONE**

**A Public Health Community Health Worker-Delivered Intervention to Reduce Human Trafficking Among Denotified Tribes in India: A Protocol Paper**

--Manuscript Draft--

| **Manuscript Number:** | PONE-D-24-33545 |
| --- | --- |
| **Article Type:** | Registered Report Protocol |
| **Full Title:** | A Public Health Community Health Worker-Delivered Intervention to Reduce Human Trafficking Among Denotified Tribes in India: A Protocol Paper |
| **Short Title:** | A Community Health Worker-Delivered Intervention to Reduce Human Trafficking Among Denotified Tribes in India |
| **Corresponding Author:** | Meredith Dank  New York University  BROOKLYN, NEW YORK UNITED STATES OF AMERICA |
| **Keywords:** | human trafficking, public health, India, community-led |
| **Abstract:** | The objective of this study is to evaluate an intervention designed to reduce human trafficking among Denotified Tribes (DNTs) in two regions of India. We will conduct a cluster-randomized controlled trial utilizing a participatory-designed, community health worker (CHW)-delivered public health intervention. CHWs will be trained to conduct anti-human-trafficking advocacy and psychological first aid (humane, supportive and practical assistance to people who are distressed) to DNT community members, and mobilize resources to ensure access to health and mental health services, education, livelihoods, and government benefits. This project leverages known effective, systemic, and sustainable approaches to reducing vulnerabilities to trafficking among DNT communities, through increased economic alternatives, health and mental health services guided by the trafficking-survivor-informed treatment protocols. |
| **Order of Authors:** | Meredith Dank |
|  | Sheldon Zhang |
|  | Stephen Abeyta |
|  | Lauren Moton |
|  | Hanni Stoklosa |
|  | Nani Cuadrado |
|  | Alex Dahlen |
|  | David J. Farabee |
| **Additional Information:** |  |
| **Question** | **Response** |
| **Financial Disclosure**  Enter a financial disclosure statement that describes the sources of funding for the work included in this submission. Review the [submission guidelines](http://journals.plos.org/plosone/s/submission-guidelines#loc-financial-disclosure-statement) for detailed requirements. View published research articles from [*PLOS ONE*](https://journals.plos.org/plosone/) for specific examples.  This statement is required for submission and **will appear in the published article** if | Yes |

*Powered by Editorial Manager® and ProduXion Manager® from Aries Systems Corporation*

| the submission is accepted. Please make sure it is accurate. |  |
| --- | --- |
| **Funded studies**  Enter a statement with the following details:   - Initials of the authors who received each award - Grant numbers awarded to each author - The full name of each funder - URL of each funder website - Did the sponsors or funders play any role in the study design, data collection and analysis, decision to publish, or preparation of the manuscript? |  |
| Did you receive funding for this work? |  |
| Please add funding details.  as follow-up to "**Financial Disclosure**  Enter a financial disclosure statement that describes the sources of funding for the work included in this submission. Review the [submission guidelines](http://journals.plos.org/plosone/s/submission-guidelines#loc-financial-disclosure-statement) for detailed requirements. View published research articles from [*PLOS ONE*](https://journals.plos.org/plosone/) for specific examples.  This statement is required for submission and **will appear in the published article** if the submission is accepted. Please make sure it is accurate. | This project was funded by the U.S. State Department (Grant # SSJTIP22CA0034). The opinions, findings and conclusions stated herein are those of the authors and do not necessarily reflect those of the United States Department of State. |
| **Funded studies**  Enter a statement with the following details:   - Initials of the authors who received each award - Grant numbers awarded to each author - The full name of each funder - URL of each funder website - Did the sponsors or funders play any role in the study design, data collection and analysis, decision to publish, or preparation of the manuscript? |  |
| Did you receive funding for this work?" |  |
| Please select the country of your main | UNITED STATES – US |

*Powered by Editorial Manager® and ProduXion Manager® from Aries Systems Corporation*

| research funder (please select carefully as in some cases this is used in fee calculation).  as follow-up to "**Financial Disclosure**  Enter a financial disclosure statement that describes the sources of funding for the work included in this submission. Review the [submission guidelines](http://journals.plos.org/plosone/s/submission-guidelines#loc-financial-disclosure-statement) for detailed requirements. View published research articles from [*PLOS ONE*](https://journals.plos.org/plosone/) for specific examples.  This statement is required for submission and **will appear in the published article** if the submission is accepted. Please make sure it is accurate. |  |
| --- | --- |
| **Funded studies**  Enter a statement with the following details:   - Initials of the authors who received each award - Grant numbers awarded to each author - The full name of each funder - URL of each funder website - Did the sponsors or funders play any role in the study design, data collection and analysis, decision to publish, or preparation of the manuscript? |  |
| Did you receive funding for this work?" |  |
| **Competing Interests**  Use the instructions below to enter a competing interest statement for this submission. On behalf of all authors, disclose any [competing interests](http://journals.plos.org/plosone/s/competing-interests) that could be perceived to bias this work—acknowledging all financial support and any other relevant financial or non- financial competing interests.  This statement is required for submission and **will appear in the published article** if the submission is accepted. Please make sure it is accurate and that any funding sources listed in your Funding Information | The authors have declared that no competing interests exist. |

*Powered by Editorial Manager® and ProduXion Manager® from Aries Systems Corporation*

| later in the submission form are also declared in your Financial Disclosure statement.  View published research articles from  [*PLOS ONE*](http://journals.plos.org/plosone/) for specific examples. |  |
| --- | --- |
| **NO authors have competing interests**  Enter: *The authors have declared that no competing interests exist.*  **Authors with competing interests**  Enter competing interest details beginning with this statement:  *I have read the journal's policy and the authors of this manuscript have the following competing interests: [insert competing interests here]* |  |
| * typeset |  |
| **Ethics Statement**  Enter an ethics statement for this submission. This statement is required if the study involved:   - Human participants - Human specimens or tissue - Vertebrate animals or cephalopods - Vertebrate embryos or tissues - Field research   Write "N/A" if the submission does not require an ethics statement.  General guidance is provided below. Consult the [submission guidelines](http://journals.plos.org/plosone/s/submission-guidelines#loc-guidelines-for-specific-study-types) for detailed instructions. **Make sure that all information entered here is included in the Methods section of the manuscript.** | New York University Approval Number:  IRB-FY2023-7128  Oral consent was obtained |

*Powered by Editorial Manager® and ProduXion Manager® from Aries Systems Corporation*

| **Format for specific study types**  **Human Subject Research (involving human participants and/or tissue)**   - Give the name of the institutional review board or ethics committee that approved the study - Include the approval number and/or a statement indicating approval of this research - Indicate the form of consent obtained (written/oral) or the reason that consent was not obtained (e.g. the data were analyzed anonymously)   **Animal Research (involving vertebrate animals, embryos or tissues)**   - Provide the name of the Institutional Animal Care and Use Committee (IACUC) or other relevant ethics board that reviewed the study protocol, and indicate whether they approved this research or granted a formal waiver of ethical approval - Include an approval number if one was obtained - If the study involved *non-human primates*, add *additional details* about animal welfare and steps taken to ameliorate suffering - If anesthesia, euthanasia, or any kind of animal sacrifice is part of the study, include briefly which substances and/or methods were applied   **Field Research**  Include the following details if this study involves the collection of plant, animal, or other materials from a natural setting:   - Field permit number - Name of the institution or relevant body that granted permission |  |
| --- | --- |
|  |  |
| **Data Availability**  Authors are required to make the data underlying their research findings fully available, without restriction.  If data need to be collected, modified or processed specifically for your study, or if participants need to be recruited specifically for your study, then it should | No - some restrictions will apply |

*Powered by Editorial Manager® and ProduXion Manager® from Aries Systems Corporation*

| occur only after your Registered Report Protocol is accepted for publication.  If the manuscript reports pilot results, the data underlying those results should be made available at the time of publication. Submission of this Registered Report Protocol implies a commitment that the data collected in the study, upon completion, will be made available with the Registered Report publication.  When legal or ethical requirements prohibit public data sharing, authors must indicate how researchers may obtain access to the data. See the [PLOS Data](https://journals.plos.org/plosone/s/data-availability) [Policy](https://journals.plos.org/plosone/s/data-availability) and [FAQ](https://journals.plos.org/plosone/s/data-availability#loc-faqs-for-data-policy) for detailed information.  A Data Availability Statement describing where the data can be found is required at submission. Your answers to this question constitute the Data Availability Statement and **will be published in the article**, if accepted. |  |
| --- | --- |
| **Important:** Stating ‘data available on request from the author’ is not sufficient. If your data are only available upon request, select ‘No’ for the first question and explain your situation in the text box. |  |
| Do the authors confirm that all data underlying the findings of the study outlined in this Registered Report Protocol will be made fully available without restriction? |  |
| **Describe where the data may be found in full sentences. If you are copying our sample text, replace any instances of XXX with the appropriate details.**   - If **no pilot data are reported in the manuscript**, please enter the following: *All relevant data from this study will be made available upon study completion.* | All relevant data from this study will be made available upon study completion. |

*Powered by Editorial Manager® and ProduXion Manager® from Aries Systems Corporation*

| - If the manuscript reports pilot data **held in a public repository**, include URLs, accession numbers or DOIs. If this information will only be available after acceptance, indicate this by ticking the box below. For example: *All XXX files are available from the XXX database (accession number(s) XXX, XXX.).* - If the manuscript reports pilot data contained **within the manuscript and/or Supporting Information files**, enter the following:   *All relevant data are within the manuscript and its Supporting Information files.*   - If neither of these applies but you are able to provide **details of access elsewhere**, with or without limitations, please do so. For example:   *Data cannot be shared publicly because of [XXX]. Data are available from the XXX Institutional Data Access / Ethics Committee (contact via XXX) for researchers who meet the criteria for access to confidential data.*  *The data underlying the results presented in the study are available from (include the name of the third party and contact information or URL).*   - This text is appropriate if the data are owned by a third party and authors do not have permission to share the data.   * typeset |  |
| --- | --- |
| Additional data availability information: | Tick here if the URLs/accession numbers/DOIs will be available only after acceptance of the manuscript for publication so that we can ensure their inclusion before publication. |

*Powered by Editorial Manager® and ProduXion Manager® from Aries Systems Corporation*

Manuscript	[Click here to access/download;Manuscript;PEMS Protocol Paper_Final Draft_07Aug2024.docx](https://www2.cloud.editorialmanager.com/pone/download.aspx?id=37165582&guid=b5ee0380-dd20-4e0f-b4b3-a3a1a40b1dcc&scheme=1)

1

2

# 3 A Public Health Community Health Worker-Delivered Intervention to Reduce Human

4 **Trafficking Among Denotified Tribes in India: A Protocol Paper**

5

6

7 Meredith Dank^1*^ , Sheldon Zhang^2^, Stephen Abeyta^1^ , Lauren Moton^1^, Hanni Stoklosa^3^, Nani Cuadrado^4^, Alex Dahlen^1^
8 David J. Farabee^5^

9

10

11 1. New York University
12 2. University of Massachusetts, Lowell
13 3. Department of Medicine. Brigham and Women’s Hospital
14 4. HEAL Trafficking
15 5 New York University Grossman School of Medicine

16

17 * Corresponding author
18 Email: [mdank@nyu.edu](mailto:mdank@nyu.edu)

19

## 20 Abstract

21 The objective of this study is to evaluate an intervention designed to reduce human trafficking among Denotified Tribes (DNTs) in two
22 regions of India. We will conduct a cluster-randomized controlled trial utilizing a participatory-designed, community health worker (CHW)-
23 delivered public health intervention. CHWs will be trained to conduct anti-human-trafficking advocacy and psychological first aid (humane,
24 supportive and practical assistance to people who are distressed) to DNT community members, and mobilize resources to ensure access to health
25 and mental health services, education, livelihoods, and government benefits. This project leverages known effective, systemic, and sustainable
26 approaches to reducing vulnerabilities to trafficking among DNT communities, through increased economic alternatives, health and mental health
27 services guided by the trafficking-survivor-informed treatment protocols.

## 28 Introduction

29 India has 1,500 nomadic and semi-nomadic tribes and 198 denotified tribes. These DNTs make up 10% of India’s population (Idate,
30 2017). Under the Criminal Tribes Act of 1871, these entire communities were stated to be criminal by nature and birth. Despite the repeal of the
31 Act in 1949 and the “denotification” of former “criminal tribes” in 1952, the DNTs continue to experience stigma and systemic oppression. For
32 example, eighty-nine percent of this population are landless and have little access to government-funded subsidies, which are tied to residence
33 (Vanavil, 2023). DNT members are deprived not only of access to social services, but they are also deprived of fundamental rights of citizenship.
34 For instance, government authorities require a Caste certificate before citizens can apply for health, education, or other social service assistance.
35 Before such a certificate is granted, the applicant needs to provide proof of residence, the caste certificate of relatives, and a birth certificate. These
36 requirements are onerous for DNT members, who may not have the required documents and face disinterest from government workers (Idate,

37	2017; Vanavil, 2023).

38 To sustain their livelihood, DNTs migrate from state to state and eke out a living from seasonal farming, domestic work, acrobatics,
39 dancing, snake charming, hunting, fortune-telling, brewing liquor, begging, handicrafts, and construction work. Low-skilled laborers find
40 themselves victims of physical and verbal abuse, coercion and forced labor practices. Chronic poverty, illiteracy, unemployment, health
41 complications, and substandard living conditions compound their vulnerability and can keep DNT families in subjugation. Additionally, of the 198
42 DNT communities in India, six to ten tribes are known to participate in intergenerational commercial sex where this work became the tribe’s
43 primary source of livelihood (Idate, 2017). The combination of abject poverty and common participation in the commercial sex can create
44 conditions in which DNT members become exploited often rising to the level of trafficking.
45 Human trafficking among DNTs has been overlooked by anti-trafficking efforts and research. The DNT community identifies the
46 following gaps in anti-trafficking response: lack of access to health care, government benefits, alternative livelihood pathways, and harm reduction
47 tools. Lack of access to government benefits and alternative livelihood pathways means DNT members have limited options to support their
48 families and are often exploited for their labor. Many lack skills, awareness, and opportunities to prevent force, fraud, or coercion in the
49 workplace. Many factors drive these gaps, including stigma faced by DNTs, their lack of trust in external resources and ability to pursue
50 alternative livelihood pathways, lack of political will, and a lack of DNT community advocates’ ability to build community members' resiliency.
51 Poverty and exploitation in the DNT communities is seemingly accepted, both by DNTs and broader Indian society, and attempts to change the
52 social status of DNTs and their access to resources often fail.
53 Although recent actions have tried to correct discrimination toward the DNT community, many DNTs are unaware of or unable to access
54 their entitled benefits. One study documented DNT members refusing to access benefits, such as children’s education, due to negative responses
55 from school administrators, teachers, and other students (Madane, 2016). India’s DNT special commission, established in 2005, has had limited
56 impact on the plight of DNTs. Society’s general suspicion of, direct aggression towards, and absence of any legal protection for DNTs are
57 ongoing. There is also a stigma against those engaged in commercial sex and victims of sex trafficking in India, reinforcing the perception of DNT
58 members as “less than.”
59 The present study seeks to address these gaps by first reporting the baseline experiences of DNTs in two Indian districts, one in the state of
60 Madhya Pradesh and the other in Tamil Nadu. This baseline study will then inform and create transformational change through the development of
61 a Community Health Worker (CHW)-delivered public health, community-based participatory designed intervention.

## 62 Materials and methods

63 **Intervention**
64 The intervention seeks to address different DNT vulnerabilities to trafficking through psychological first aid and supported referrals that
65 are guided by the trafficking-survivor-informed, evidence-based, Privacy Educate Ask Respect Respond (PEARR) Tool and (Health, Education,
66 Advocacy, Linkage) HEAL Protocol Toolkit (Baldwin et al., 2017). The PEAAR approach’s goal is to have informative conversations with
67 patients that promote health, safety, and wellbeing in safe environments that encourage patients to open and accept further services. The project
68 will hire and train two types of community health workers (CHWs): Community Mobilizers (CM) and Health Fellows (HF). The CMs will be
69 responsible for community mobilization and ensuring access to education, livelihoods, and entitlements. HFs will be responsible for health access
70 activities. CMs and HFs will be trained in psychological first aid counseling and trafficking.
71 At the core of our harm-reduction counseling are motivational interviewing and trauma-informed safety planning that help participants
72 develop resilience and empower them to navigate life challenges and secure gainful employment free from exploitation. For our supported
73 referrals, we will utilize the survivor informed and evidence based HEAL Protocol Toolkit that is used in over 35 countries to craft minimum
74 standards for trafficking response in health care settings (Baldwin et al., 2023). The CMs will facilitate participants’ contact with trafficking victim
75 service advocates who can provide legal advice, alternative incomes, vocational training access, and healthcare access.
76 Within each treatment hamlet, a Community Awareness and Action Group (CAAG) will be formed and participatory mapping methods
77 will be implemented to select community actions and review intervention performance. The goal is to connect community members to health care,
78 counseling, education opportunities, government resources (such as caste-based entitlements), and alternative pathways to livelihood in order to
79 reduce trafficking in these communities. To improve access to government-sponsored entitlements and social security programs, we will train
80 CMs on key provisions of social security programs and application procedures. They will develop community awareness of programs and kiosks
81 (a location within the hamlet where the CM will assist the community member with registering for government schemes) to assist members in
82 submitting applications. Their program activities will be informed by the community level mapping of key issues and aspirations related to social
83 security programs. The CHFs and CAAG will work together in all aspects of the program, review performances and adjust activities accordingly.
84 Ongoing training and supervision by the research team will support CHFs and maximize their ability to engage and assist intervention participants.

## 85 Impact evaluation

86 We will use a cluster-randomized trial (CRT) to evaluate the impact of the proposed intervention. We will use household- and individual-
87 level survey measures to evaluate work exploitation and human trafficking risk factors. We will compare the hamlets that receive our intervention
88 to a set of matched control hamlets, as follows.
89 *Pairing of Eligible Hamlets Prior to Randomized Assignment*
90 Intervention and matched hamlets were chosen in a three-step process. First, DNT hamlets were screened for eligibility and logistical
91 feasibility. This resulted in 62 eligible hamlets in the project site in Tamil Nadu and 30 in the project site in Madhya Pradesh. Second, the eligible
92 hamlets were clustered based on demographic and socio-economic factors that were measured in our initial community mapping exercise.
93 Specifically, clusters were formed using the following features: the number of households, gender breakdown, number of children, caste
94 certifications obtained, and general household income. Similarity scores were based on Gower similarity scores. We identified 10 clusters of
95 hamlets in the project site in Tamil Nadu and 6 clusters in the project site in Madhya Pradesh. Third, pairs of hamlets within each cluster were
96 identified and screened by our in-country partner to ensure that no pairs were physically adjacent to one another, to prevent possible crossover
97 contaminations. Pairs were randomly selected for the study, and hamlets within each pair were randomly assigned to the intervention group or to
98 control group. Table 1 shows the resulting pairs of hamlets in our study.
99 *Participant Screening*
100 We applied a household-based census approach to screen all eligible households in these selected hamlets. Based on census data (one site
101 had 217 households in the treatment group and the other had 166) and community mapping, our field partner built an exhaustive list of all
102 households in the selected hamlets. Upon arrival at a DNT hamlet, our team of enumerators used the list of all dwelling units to initiate the data
103 collection process. Within each household, we identified a single eligible participant who was between the ages of 18-55 to complete the survey.
104 If a household had more than one eligible participant, we applied the birthdate scheme to select whoever had the birthdate closest to the
105 enumerator visit date. If all eligible respondents were absent at the time of the field visit but were still living in the household (i.e., had not
106 migrated elsewhere), information would be sought on when the individual would return and up to two additional attempts were made. All
107 households in the selected hamlets were contacted, and our recruitment procedure ensured all eligible members of each hamlet would have equal
108 chances of being included in the study.
109 The baseline survey (which has already occurred) was administered on a computer tablet and lasted anywhere between 45-60 minutes.
110 Consent was obtained at beginning of the survey, with the following information explained to the participants:
111 1. The survey was voluntary, participant may stop at any time.
112 2. Participation or non-participation will not affect relationships with our field partner.
113 3. Information would be kept confidential. Will be assigned unique ID and responses would not be shared except in aggregate.
114 4. Participant may stop the interview at any time.
115 5. Enumerators would confirm that participant understood their rights and had no questions prior to the commencement of the survey
116 interview.
117 6. Enumerators were to conduct the interviews in a space where conversation could not be heard by others.

## 118 Research questions and hypotheses

119 The main research question in this project is whether a community-based participatory intervention will empower the DNT communities
120 to increase economic opportunities and access to government entitlements, thus reducing the collective risk exposure to human trafficking. We
121 seek to apply a clustered randomized trial (CRT) and a rigorous Monitoring/Evaluation/Research/Learning (MERL) process to ensure a high
122 fidelity in implementation of the proposed intervention. Further, this project has the following hypotheses, that relative to the control hamlets, in
123 reducing at a greater rate individual-level risk of trafficking or/and experiences of trafficking violations:
124 (1) HFs recruited from treatment DNT hamlets and trained to deliver a multi-pronged intervention, including psychological first aid,
125 community health interventions in collaboration with the CAAGs.
126 (2) CMs recruited from treatment DNT hamlets and trained will empower their communities to access more entitlements and social
127 security programs, through connecting community members in participating areas to health care resources, governmental resources, and alternative
128 pathways to livelihood.
129 (3) Residents of the treatment hamlets will report having more alternative livelihood opportunities, and more success in entrepreneurship
130 and economic innovations.
131 (4) Residents in the treatment hamlets will report having greater access to basic literacy and secondary education opportunities as a result
132 of tutoring, transportation and application assistance.
133 (5) Fewer residents of the treatment hamlets will be victimized by workplace exploitations and trafficking violations.

## 134 Study organization and sites

135 This project is a collaboration between New York University, HEAL Trafficking, and our local Indian partner agency. The study sites are
136 hamlets located in the State of Madhya Pradesh and the State of Tamil Nadu, India. At the request of our local partner, we have decided not to
137 name the specific study sites and our local implementation agency due to political sensitivity.

## 138 Regulatory affairs and data and safety monitoring

139 This study protocol was reviewed and approved by the New York University Institutional Review Board. IRB-FY2023-7128. The protocol
140 was registered with the Evidence in Governance and Politics (EGAP) registry (ID: 20230925AA).
141 **Participants**	In all, 790 households (one adult participant surveyed from each household) were enrolled in the evaluation, including 382 (48%)
142 from the treatment hamlets and 408 (52%) from the control hamlets. Of these household heads, 44% were male. Most of these participants were
143 between 26 and 40 years old. Twenty-five percent had received at least 9 years of formal education; 63% reported being married at the time of the
144 survey, and 47% reported that they did not have a caste certificate.

## 145 Assessments

146 To assess baseline rates and to evaluate our impact, we designed a survey instrument to quantify the prevalence of trafficking-prone
147 activities and trafficking victimization. We also produced a short version of the survey which can be completed in 10-15 minutes for monitoring
148 purposes. The longer survey will be administered at baseline and endline in all hamlets; the shorter, monitoring survey will be administered
149 quarterly in the intervention hamlets.
150 We applied two strategies to establish baseline measurement to gauge changes in human trafficking in this population and intervention-
151 outcome: (1) prevalence in trafficking-prone activities, namely employment and livelihoods, in these DNT hamlets, and (2) prevalence in
152 trafficking victimization among these hamlets. The intent is to measure changes in both the extent of participation by DNT hamlets in trafficking-
153 prone activities and their actual human-trafficking victimization. Once the baseline is established, we will repeat the survey at the endline to
154 compare changes between the two clusters over time. A list of targeted outcomes for the project is shown in Table 2.

## 155 Survey Instrumentation

156 This project built an instrument that assesses the following domains from respondents: (1) demographic profiles of the respondent and
157 his/her family members; (2) employment activities, (3) work and earnings, (4) exploitative practices, (5) rights violations, and (6) engagement with
158 services, law enforcement. The surveys also included the Short Form-12 (Ware et al., 1996), along with measures of trafficking experiences
159 (OHTS; Cutbush et al., 2023), healthcare access (Tessema et al., 2022), healthcare spending (Heijink et al. 2011), and stages of change (adapted
160 from the URICA; Twis et al., 2022). A copy of the baseline survey is included as Supplementary Material. A copy of the baseline survey is
161 included as Supplementary Material.

## 162 Treatment conditions

163 The study design involves random assignment of hamlets to an early or delayed intervention condition. This allows us to assess the impact
164 of the intervention while ensuring that all randomized participants will eventually receive it.

## 165 Statistical analysis plan

166 This evaluation will rely on a cluster-randomized trial occurring in 23 hamlets: 11 in a project site in the state of Madhya Pradesh and 12
167 in a project site in the state of Tamil Nadu. Study participants in the intervention group will respond to a quarterly survey over the course of the
168 study to assess receipt of the proposed intervention activities, and all study participants will participate in a more in-depth baseline and follow-up
169 interview. The latter will allow for the direct assessment of the intervention’s impact and will also provide a wide range of covariates. Based on
170 the baseline survey, the percentages of those reporting exploitation (the primary outcome) in the preceding five years was similar between study
171 conditions (Test hamlets: 57.3% ± 5.0% vs Control hamlets: 61.9% ± 4.7%).
172 *Analytic approach*. Our primary statistical approach will be a matched difference-in-differences comparisons of the rates of work exploitation
173 between test and control hamlets. We will compare the average change between the two timepoints in the test hamlets to the control hamlets. In
174 addition to overall exploitation, we will consider the secondary outcomes in Table 2. In addition, we will analyze the quarterly monitoring survey
175 using a longitudinal, multi-level mixed-effects logistic model of the form logit(E(exploitation)) = ∑ 𝛽 × Time Point + 𝜆𝑖 + 𝜂𝑗, where 𝜆𝑖 is a
176 person-level random intercept to account for the multiple time periods, and 𝜂𝑗 is a hamlet-level random intercept to account for clustering. For
177 both analysis methods, we will consider a test for heterogenous treatment effects by site (Madhya Pradesh v. Tamil Nadu) using interaction terms.
178 *Missing data*. The following measures were taken to minimize missing data: If a study participant is not reached the first time, the field
179 staff will follow up at least one more time, including by texting/calling. If a study participant still cannot be reached, the field staff will ask
180 someone else in the household to fill it out for them. The research team will regularly check the missingness levels for each question. If any
181 question has a high rate of being skipped, the team will investigate and consider rewriting the question.
182 Despite these strategies, some missing data is inevitable. Our primary strategy will be to use MICE with 10 dataset replications, using
183 Rubin’s rules to pool point estimates and standard errors. As a sensitivity analysis, we will also report: 1) the complete case result; 2) the worst-
184 case result, where missing values are imputed as y=1 indicating exploitation; 3) the best-case result, where missing values are imputed as y=0,
185 indicating no exploitation.

## 186 Discussion

187 Historical discrimination against members of DNTs has resulted in limited access to critical services, including healthcare, education, and
188 basic government services like obtaining documentation or land titles. As a result, DNT members are at greater risk of maltreatment, exploitation
189 and trafficking. Using an intent-to-treat cluster randomized trial, this project will assess the effectiveness of an intervention designed to address
190 these service gaps for this population. The results of the final evaluation are expected in fall of 2027.

## 191 Contributions

192 Meredith Dank-- Conceptualization, funding acquisition, and project administration.
193 Sheldon Zhang—Conceptualization, funding acquisition, and project administration.
194 Stephen Abeyta—M&E MonitoringLauren Moton—Project administration
195 Hanni Stoklosa—Project administration
196 Nani Cuadrado—Project administration
197 Alex Dahlen—Formal analysis
198 David Farabee—Project administration

## 199 Limitations

200 Despite the use of a randomized design and established survey measures, this evaluation has at least three important limitations. First, the
201 outcomes measures rely on self-reports, which can be problematic when assessing sensitive behaviors ). Second, given that the intervention may
202 raise respondents’ awareness of exploitation, it is possible that participation could increase *reported* levels of exploitation, even if such events
203 remained stable. One approach to address the latter limitation will be to focus on exploitative conditions and *events*, rather than self-reported
204 perceptions of the crime. Lastly, due to the lack of livelihood opportunities in and around the treatment hamlets, outward household migration (i.e.,
205 case attrition) may become an issue during the intervention period. We may apply statistical procedures to impute outcomes for lost participants,
206 although we assume that the attrition rates will be similar across both treatment and control hamlets due to similar broader socio-economic
207 environments. In addition, we will conduct a sensitivity analysis comparing outcomes using the imputed values versus outcomes obtained by
208 treating values from missing participants as missing.

## 209 Acknowledgements

210 This project was funded by the U.S. State Department (Grant # SSJTIP22CA0034). The opinions, findings and conclusions stated herein are
211 those of the authors and do not necessarily reflect those of the United States Department of State. We are grateful for our local staff based in Tamil
212 Nadu and Madhya Pradesh for enumerating the households in the participating hamlets and conducting the surveys for this project. We are also
213 grateful to the residents of the participating hamlets for providing input in the development of the intervention.

## 214 References

215 Baldwin S.B., Barrows J., Stoklosa H. (2017). Protocol Toolkit for Developing a Response to Victims of Human Trafficking. HEAL Trafficking
216 and Hope for Justice.
217 Baldwin, S. B., Miller, C. L., Maclin, B. J., Williams, S., & Stoklosa, H. (2023). Toward an Evidence-Based Framework to Guide Health Care
218 Responses for Patients Impacted by Human Trafficking: Evaluation of the HEAL Trafficking Protocol Toolkit . *Journal of Human*

219	*Trafficking*, 1–15. https://doi.org/10.1080/23322705.2023.2170693

220 Cutbush, S., Charm, S., Tueller, S., Krieger, K. and Gibbs, D., 2023. The Outcomes for Human Trafficking Instrument: validity and reliability
221 testing. *Journal of human trafficking*, *9*(3), pp.299-310.
222 Heijink, R. Priyanka Saksena, K, & Evans. D. (2011). *Validity and Comparability of Out-of-pocket Health Expenditure from Household Surveys:*
223 *A review of the literature and current survey instrument*. Geneva: WHO.
224 Idate, B. R. (2017). *National Commission for Denotified Nomadic and Semi-Nomadic Tribes. Ministry of Social Justice and Empowerment.*
225 <https://socialjustice.gov.in/writereaddata/UploadFile/Idate%20Commission.pdf>
226 Madane, U. (2016). Social Exclusion and Inclusion of De-Notified, Nomadic and Semi-Nomadic Tribes in India. *Asian Journal of Research in*
227 *Social Sciences and Humanities*, *6*(9), pp. 1726-1736.
228 RTI International. (2020). *Outcomes for human trafficking survivors: User guide*. Research Triangle Park, NC.
229 Stewart AL, Ware JE, editors. *Measuring functioning and well-being: the medical outcomes study approach*. Duke University Press; 1992.
230 Tessema ZT, Worku MG, Tesema GA*, et al* Determinants of accessing healthcare in Sub-Saharan Africa: a mixed-effect analysis of recent
231 Demographic and Health Surveys from 36 countries *BMJ Open* 2022;12:e054397. doi: 10.1136/bmjopen-2021-054397.

| 232 | Twis, M.K., Cimino, A. and Files, M., 2022. Does individual advocacy work?: A research and evaluation protocol for a youth anti-sex trafficking |
| --- | --- |
| 233 | program. *PLoS one*, *17*(6), p.e0270103. |
| 234 | Vanavil Trust (2023). *Status of Nomadic Tribes in Tamil Nadu: Participatory Survey Report*. |
| 235 | Ware J Jr, Kosinski M, Keller SD. A 12-Item Short-Form Health Survey: construction of scales and preliminary tests of reliability and validity. |
| 236 | Med Care. 1996 Mar;34(3):220-33. doi: 10.1097/00005650-199603000-00003. PMID: 8628042. |
| 237 |  |
| 238 | **Table 1:** *Randomized Clusters of Treatment and Control Hamlets** |

## Treatment

**Num.**

## Households Control

**Num.**

## Households

| **Madhya Pradesh Site:** |  | | | |
| --- | --- | --- | --- | --- |
| Tx_Hamlet1 | 15 | Ctrl_Hamlet1 |  | 20 |
| Tx_Hamlet2 | 18 | Ctrl_Hamlet2 |  | 19 |
| Tx_Hamlet3 | 6 | Ctrl_Hamlet3 |  | 18 |
| Tx_Hamlet4 | 95 | Ctrl_Hamlet4 |  | 120 |
| Tx_Hamlet5 | 26 | Ctrl_Hamlet5 |  | 70 |
| Tx_Hamlet6 | 90 | NA |  |  |
| *Total households* | *250* |  | *Total households* | *247* |
| **Tamil Nadu Site:** |  |  |  | |
| Tx_Hamlet1 | 35 | Ctrl_Hamlet1 | 41 | |
| Tx_Hamlet2 | 39 | Ctrl_Hamlet2 | 58 | |
| Tx_Hamlet3 | 47 | Ctrl_Hamlet3 | 39 | |
| Tx_Hamlet4 | 37 | Ctrl_Hamlet4 | 36 | |
| Tx_Hamlet5 | 48 | Ctrl_Hamlet5 | 23 | |
| Tx_Hamlet6 | 15 | Ctrl_Hamlet6 | 35 | |

*Total households	221	Total households	232*

239	*Hamlet names are anonymized at the request of local implementation partner due to political sensitivity.

240

241	**Table 2**: *Outcome Metrics (Collected Quarterly)*

## Category Outcome Source

Exploitation/Trafficking

Primary outcome	(1) % who experienced at least one type of workplace exploitation

(2) % who experienced enough workplace exploitations/abuses to be considered potential victims of human trafficking

Endline and quarterly surveys

Secondary outcomes	% of participants involved in other at-risk/illicit economic activities	Endline and quarterly surveys

Risk Factors

% experiencing trafficking victimization or participating in trafficking- prone activities

Endline and quarterly surveys

Employment type	% of respondents engaged in commercial sex in any capacity	Endline and quarterly surveys

Employment type	% of participants who have or secure a living wage through a non- exploitative/illicit job during the project

Schooling	% of children in the household going to school / who have dropped out of school

Endline and quarterly surveys Endline and quarterly surveys

Healthcare	% who have seen a health care worker in their hamlet	Endline and quarterly surveys

Government	(1) #/% of participants who have obtained or applied to social security or other government services

Endline and quarterly surveys

(2) #/% of participants who have obtained or applied to entitlement documents	Endline and quarterly surveys
(3) % with a caste certificate	Endline and quarterly surveys
(4) % who worked under MGNREGA (Mahatma Ghandi National Rural	Endline and quarterly surveys

Employment Guarantee Act)

242

IRB Approval Letter

[Click here to access/download](https://www2.cloud.editorialmanager.com/pone/download.aspx?id=37476859&guid=6ae17942-bcdf-4dc4-ad13-7c104e0aebaf&scheme=1)

[**Supporting Information** IRB.pdf](https://www2.cloud.editorialmanager.com/pone/download.aspx?id=37476859&guid=6ae17942-bcdf-4dc4-ad13-7c104e0aebaf&scheme=1)
