# Supplementary material for: A public health community health worker-delivered intervention to reduce human trafficking among Denotified Tribes in India: A protocol paper
Source: PLoS One. 2025 Mar 13;20(3):e0317733. doi: 10.1371/journal.pone.0317733 (PMC11906080; doi:10.1371/journal.pone.0317733)
Supplement: Attachment — Submitted filename: Response to reviewers.docx [file pone.0317733.s003.docx]

**November 20, 2024**

Kasi Eswarappa
Academic Editor
PLOS ONE

RE: PONE-D-24-33545

**Dear Dr. Eswarappa:**

Thank you for sending the comments regarding to our manuscript “A Public Health Community Health Worker-Delivered Intervention to Reduce Human Trafficking Among Denotified Tribes in India: A Protocol Paper.” We were pleased with the positive reviews and have revised the manuscript to address the reviewers’ concerns. These revisions are summarized in italics below, and we have uploaded both marked and clean copies of the revised manuscript.

**Reviewer #1**

- The protocol refers broadly to the term "human trafficking," but that term has different interpretations in different nations and contexts. Please specify inclusion/exclusion descriptions and procedures. For instance: What about extended family members who willingly engage in sex work or physical labor that would appear from an American perspective to be exploitative, but the local norms and legal codes accept such arrangements?

*Response: Thank you for raising this important definitional issue. Because of our funding source, this study adopts the term “human trafficking” as legally defined by the US Trafficking Victims Protection Act of 2000 and operationalized by the US State Department’s Office to Monitor and Combat Trafficking in Persons through its PRIF (Prevalence Reduction Innovation Forum) program. As suggested, we have added texts to clarify the use of this terminology.*

- This study was reviewed by an IRB in the USA, but given the vast cultural differences, a review by an equivalent entity in India would be best practice, although not required.

*Response:* *Thank you for pointing out this omission. The study was reviewed and approved by an in-country research institute’s IRB. We have added this information under section “Regulatory affairs and data and safety monitoring”.*

- The word "participatory" appears throughout the manuscript, but the researchers appear to be interfacing with the community health workers and other interventionists, rather than the members of the denotified tribes being served. Since the term "participatory research" refers to those benefitting from services, rather than to interventionists from a different background, please replace all instances of the word “participatory” with more accurate terms and descriptions, such as "with the collaboration of local service providers."

*Response: Thank you for pointing out this apparent lack of clarity in our description of the personnel hired to implement the program. To be clear, all interventionists, i.e., community health workers (CHWs) and community mobilizers (CMs), are recruited directly from the targeted DNT communities. They are trained and deployed back to the communities where they came from. We have added texts to clarify this point, at the end of the Introduction section.*

- The intervention was developed in an entirely different context than the two regions of India where the study will occur. So clarify that the intervention was not developed in India for the population targeted by this study. Although an intervention developed in Europe or North America may be commonly exported for use in other countries, that does not provide evidence of validity with the local population in this study. Local context matters, with cross-cultural research optimally including steps of cultural adaptation and validation (e.g., member checks in pilot tests to confirm that the interventions are received as intended). In this protocol, please list any cultural adaptations planned/made for the denotified tribes (e.g., given how they experience fellow Indians as outsiders).

*Response: Thank you for bringing up the cross-cultural aspect to our intervention planning and implementation. This is an important point, and we have added additional texts to clarify the participatory nature of our intervention design and implementation and efforts to assess the cultural adaption of our intervention strategy, in the first paragraph under the Intervention section, and in the final paragraph under the Assessments section. Although developed in North America, the PEARR program relies heavily on a participatory process in the design and implementation of its interventions. Our in-country partner brings forward decades of field experiences and intervention know-how from working with these communities, while the North America team provides technical support in impact assessment, monitoring and evaluation design, as well as administration of the funds. Because of the current political situation where the Indian government suspects the intentions of Western-funded NGO programs, our in-country partner has requested to remove the agency name as well as the names of all participating communities to avoid unwanted attention from the authorities.*

- Since the intervention is described broadly, please specify how the intervention targets the specific outcomes evaluated. If there is an explanation for causality based on extant theory, that would be helpful to add but not essential.

*Response: due to space limitation, we have refrained from expanding our theory of change, which centers around a harm-reduction orientation through a public health vehicle and seeks to gain trust in DNT communities that have grown suspicious to outside agencies. Through a participatory process, we seek to empower DNT communities to increase access to government entitlement programs, improve awareness of health and rights protection, and develop alternative livelihoods. We have added additional texts to clarify this causal pathway in the second paragraph under the Intervention section.*

- Please specify any fidelity checks of the community health workers’ training and interventions.

*Response: Thank you for pointing out this important monitoring task. All community health workers who receive training by our trainers will be assessed through several mechanisms to ensure fidelity: (1) assessment during classroom training activities; (2) pre/post tests at the onset and conclusion of the training; 3) close oversight of our in-country collaborating agency during course of the project; and (4) our established M&E feedback loop. We provided all intervention personnel standardized forms for them to log their activities (number of people served, types of services delivered, etc.). These forms will be monitored by both the local collaborating agency as well as the NYU project director. We have added this information in the final paragraph under the Assessment section.*

- Please specify if any individuals from denotified tribes serve as cultural brokers, interfacing with the community health workers. Specify if all study personnel working with the individuals from denotified tribes are native or fluent in the same primary language - and specify the procedures for working with individuals with limited abilities to read.

*Response: this is addressed in our response to comment #3 above. All intervention personnel are members of the DNT communities. The whole intension is to build collective agency that can sustain over time.*

- Please focus the manuscript on the two regions/tribes involved in this study, rather than implying in the text that the project has relevance to all denotified tribes.

*Response: We have added this important caveat to the limitations section of the Discussion.*

- For the design, be aware that attention by community health workers will “in and of itself” modify community expectations and behavior (Hawthorne effect), so possibly consider ways to differentiate the effects of the intervention from the effects of attention and visits from outsiders.

*Response: This is an important point. It is difficult to fully mitigate the risk of a Hawthorne effect in this project, but we should note that both treatment and control hamlets have experienced increased attention from the research team and our collaborating partner as part of the initial mapping, surveying, intervention development, and baseline interviews. We have added this problem—and our attempt to address it—to the Discussion.*

**Reviewer 2**

- It will be good if you list out the tribes’ names.

*Response: Thank you for the inquiry. There* *are well known tribes that engage in commercial sex trade because of their marginalized economic status, such as Bedia, Nat, Perna, Banchara, Rajnat, Bawaria, Kanjar, etc. However, to avoid further stigmatization, we opted not to list their tribe names in any of our publications. Doing so serves no particular purposes other than publicizing which tribes we may be working with, as their general geographical locations are known to the public. For political reasons, we have also omitted specific locations where our study is taking place in this manuscript.*

- Reasons to choose these two states - Madhya Pradesh and Tamil Nadu.

*Response: Thank you for the inquiry. We have added clarifications of the factors that went into the site selection in the text, with track change on. Briefly, our study intends to achieve some geographical representation because there are distinct differences between DNT tribes in the north and south of India. Further, we had to consider hamlets with comparable sizes in population and also sufficient distances from one another to allow randomization and to minimize potential cross-contamination. Finally, we had to consider access to the physical locations of these communities.*

We are grateful for these comments and believe the revised manuscript is much-improved as a result. Please let us know if you have any further questions or comments.

Best,

Meredith Dank, PhD
